# Supplementary material for: Ni-Catalyzed Enantioselective Intramolecular Mizoroki–Heck Reaction for the Synthesis of Phenanthridinone Derivatives
Source: J Org Chem. 2023 Jun 15;88(13):8203–26. doi: 10.1021/acs.joc.3c00202 (PMC10337041; doi:10.1021/acs.joc.3c00202)
Supplement: Supplementary file 2 — jo3c00202_si_002.zip [file jo3c00202_si_002.zip › Chiral ligands/4a 6F-iQ-NO/13C 6F iQ N-O 4a/1/pdata/1/email_DR-117-078 C13_1_1.pdf]

DR-117-078 C13
C13APT CDCl3 {D:\nmrusers\malachowski} BM 6

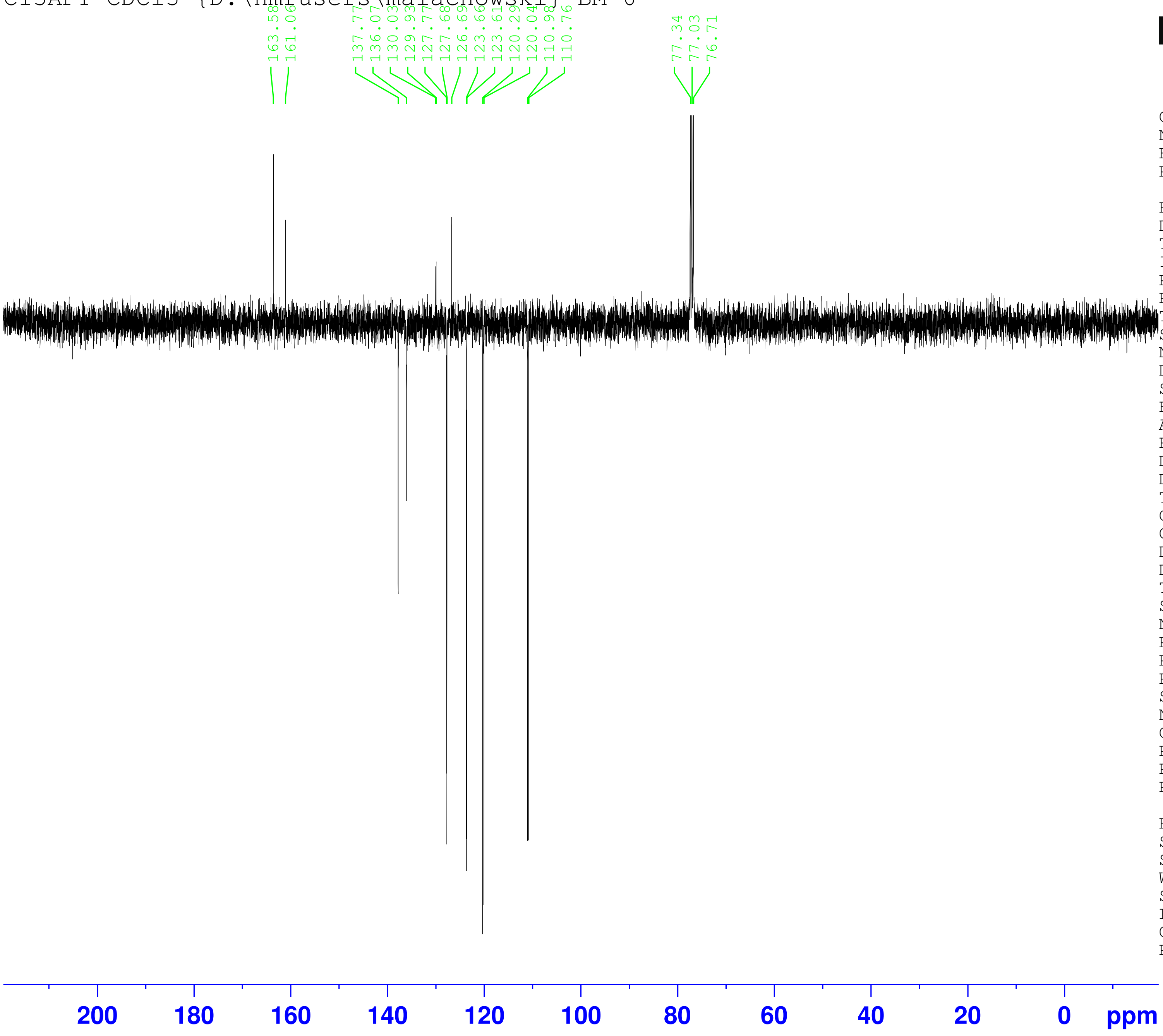

BRUKER

Current Data Parameters
NAME DR-117-078 C13
EXPNO 1
PROCNO 1

F2 - Acquisition Parameters
Date_ 20220817
Time 3.14 h
INSTRUM spect
PROBHD Z104450_0352 (
PULPROG jmod
TD 65536
SOLVENT CDCl3
NS 5000
DS 4
SWH 24038.461 Hz
FIDRES 0.733596 Hz
AQ 1.3631488 sec
RG 203
DW 20.800 usec
DE 6.50 usec
TE 298.0 K
CNST2 145.0000000
CNST11 1.0000000
D1 2.00000000 sec
D20 0.00689655 sec
TD0 1
SFO1 100.6278593 MHz
NUC1 13C
P1 8.00 usec
P2 16.00 usec
PLW1 56.13299942 W
SFO2 400.1516006 MHz
NUC2 1H
CPDPRG[2 waltz65
PCPD2 90.00 usec
PLW2 12.14200020 W
PLW12 0.24368000 W

F2 - Processing parameters
SI 32768
SF 100.6177975 MHz
WDW EM
SSB 0
LB 1.00 Hz
GB 0
PC 1.40
